# Analysis of the institutional free market in accredited medical physics graduate programs

**DOI:** 10.1002/acm2.70164

**Published:** 2025-07-14

**Authors:** Brian W. Pogue, Alexander P. Niver

**Affiliations:** ^1^ Department of Medical Physics University of Wisconsin‐Madison Madison Wisconsin USA

**Keywords:** accreditation, education, imaging, medical physics, radiation therapy

## Abstract

Medical Physics education is largely delivered through accredited programs where admission numbers and funding for students are controlled by the individual institutions providing the educational programs. Public data from these accredited graduate programs, along with funding data, can be used to analyze institutionally driven trends in the market for providing this education. Temporal trends from 2017 to 2023 show robust growth in MS graduates, increasing at an average of 17.7 per year, as compared to steady but modest growth in PhDs, increasing by 3.6 per year. The current ratio is 2:1 in MS:PhD for total annual graduates in North America. Trends in funding show self‐funding of MS students is a dominant pathway in domestic programs, with this being less dominant in international programs. Those programs dominated by accredited MS education have their largest fraction of faculty in radiation oncology departments, whereas those dominated by PhD education have their largest fraction of faculty in radiology departments. Overall, NIH funding in the space of radiation diagnostics and therapeutics has been largely static over this timeframe, but with a notable recent rise in NCI funding in the last 5 years. This can be contrasted to a substantial 5X–6X rise in NIH funding for engineering research programs during this same period, with significant increases in trainee funding there. Taken as a whole, this survey shows that growth in the field of medical physics education is dominated by MS graduates, presumably servicing the expanded growth needs for well‐trained clinical physicists. However, the research infrastructure that supports PhD training in medical physics seems likely to be growing modestly and has missed the growth trend of NIH funding to non‐accredited programs such as biomedical engineering.

## 1 INTRODUCTION

The field of Medical Physics is dominated by the 9000 members of the American Association of Physicists in Medicine (AAPM),^1^ and for 3 decades has seen continuous growth at about 200 additional members per year.^2^, ^3^, ^4^ To sustain this growth, there has been annual growth in the number of students trained in medical physics, although matching the needs and supply is an iterative challenge in the field. While the structure and content of training is administered by Commission on Accreditation of Medical Physics Education Programs, Inc. (CAMPEP),^5^ the growth in supply of trained medical physicists is actually distributed, as controlled by each participating educational institution. It is their faculty and governance structures who determine what they want to participate in, to what extent, and who gets admitted and trained. This produces a free market of education where faculty and academic administrators make strategic choices about which programs they will offer and support at their own institution and at what scale. The driving factors in education are often quite locally driven, and depend upon their department focus, finances, research capabilities and capacity, and sometimes historical experiences. Publicly available data can help assess which factors are most dominant on a national scale, providing a window into the distributed decision making at the institutional level.

The most visible influence on the field of education is the accreditation process from the CAMPEP,^6^ which formalized requirements for accredited educational programs in 2014, requiring accredited degrees for all applicants to accredited clinical residency programs.^7^, ^8^ CAMPEP is aligned with the goals of the AAPM to ensure that the field grows with well‐trained individuals.^9^, ^10^ An independent survey of trends is possible because of the CAMPEP requirement that each program post public data on their institutional website, and so this was the source of data for much of this analysis here.

The required didactic training components for accredited programs are prescribed by CAMPEP, with guidelines for admission criteria, curricula, student experience, and faculty expertise,^6^, ^10^ but still admissions and support decisions are completely dependent upon the local institutions. The choices of type of program, MS, PhD, DMP, or Certificate, and these opportunities are especially relevant for the balance of education and research, but also determine the shape of the departments that support them.

Inevitably, trends in education are affected by the methods by which student training is funded. A dominant factor at many institutions is the tradeoff in choices of how MS versus PhD students are funded, and so a study of this was conducted across institutions and as an annual aggregate trend. PhD program students are commonly funded, at least in part, by extramural support mechanisms, and so a major part of this is derived by National Institutes of Health (NIH) funding.^3^ The NIH funding data is also publicly available on their RePORTER database tool, and so a survey of this was completed to analyze available funds and some measure of successful research by funding training grant awards. The two most prestigious and competitive awards for training PhD students are the F31 Ruth L. Kirschstein Predoctoral Individual National Research Service Award,^11^ competitively awarded to individual students midway through their training, and the T32 Ruth L. Kirschstein Institutional National Research Service Award. Gaining a complete independent picture of funding of programs is a particularly challenging process because this type of data is not easily publicly available from individual programs, although some internal survey data was made available here.

Another piece of data available for this report was the faculty makeup of the program, as broken down by their primary department affiliation. On the surface, this is not particularly informative; however, aggregate numbers broken into separate groups of institutions were examined to assess trends based on departmental programs participating and how this may influence choices in MS versus PhD student numbers. Given that clinical versus academic departments may have quite different emphases or funding capabilities, this breakdown provides some insight into graduate program choices.

This aggregate pool of publicly available data provides a snapshot of the field as well as the annual trends in degrees, funding, and institutional positioning of education programs, which will all affect the field of Medical Physics. The value of this report is that it provides an independent review of this public data. Most importantly, the numbers of people educated in medical physics are driven by the free markets of individual educational institutions, and so this data can help institutions understand what the landscape is, and where their own emphasis may make the most sense, or how they could adapt to suit their own needs in the future.

## 2 METHODS

The survey of data started with the required self‐reporting totals from each documented CAMPEP graduate program, about their admits and graduates from MS, PhD, Certificate, and DMP programs. These data are required to be publicly posted on each accredited program website, and so these data were downloaded for each program. All websites were accessed in September–October 2024, and emails were sent to and received from program directors in October–November 2024. No direct requests were sent to CAMPEP. There were 59 CAMPEP accredited graduate programs found online, of which 54 had some information accessible on their websites. Within this, 22 programs did not have complete data posted, and so specific information about certain years' student numbers or faculty department information was solicited directly from the program directors by email query. A total of 14 responses were received from these 22, providing us with complete data from 51 programs, and this was the set used here. Of the programs not included, four received their accreditation in the latter years of the study (2021–2023), and so they may not have produced any graduates during the years studied. Of the remaining five, more established programs, they appeared to have a small number of students. Assuming each program graduated five students, this would mean that 9% of total graduates were not included, but this is really not possible and it is more likely that this is closer to 0 to 1% overall, so their omission would not change the aggregate trends seen here.

The individual graduate program faculty from each program is also available on their websites, and so these were aggregated in terms of which department they belonged to. There were 55 programs included in this faculty analysis, where there was ability to determine the likely home department of each faculty member. Again, for those programs that did not have a complete faculty program listing, this information was obtained through email to the program directors. The cumulative data on each home department was thought to be pertinent to understanding the overall structure of the programs that offer the CAMPEP degree.

CAMPEP Program Student support funding info was solicited by CAMPEP, and access to this survey data was granted for this research to add in cumulative information about MS and PhD student funding as an aggregate. This information is somewhat limited in value because it does not report quantitative numbers of students supported by each mechanism, but rather just lists the range of funding mechanisms possible for each program.

Finally, data on funding for research is available from the NIH in their RePORTER system, as has been previously studied,^12^ and this can be queried through the Advanced Search tab, in a number of separate ways with funding year and by institution and department. The data for Medical Physics programs cannot be queried directly, but funding for RADIATION‐DIAGNOSTIC ONCOLOGY, which is a category that combines departments of Radiology and Radiation Oncology together. This is an unfortunate grouping that cannot be easily separated out for further insight, but was used here nonetheless because it does cover the majority of departments in which CAMPEP graduate programs find themselves. The other relevant groupings to query are either Physics or Engineering, and so these were each surveyed as well. Yet, the numbers from Physics were negligible relative to Engineering. The total number of grants funded each year in each grouping was queried, as well as training grants that include T32 institutional training and F31 individual training grants.

## 3 RESULTS

### 3.1 Current enrollments across programs

**FIGURE 1:**
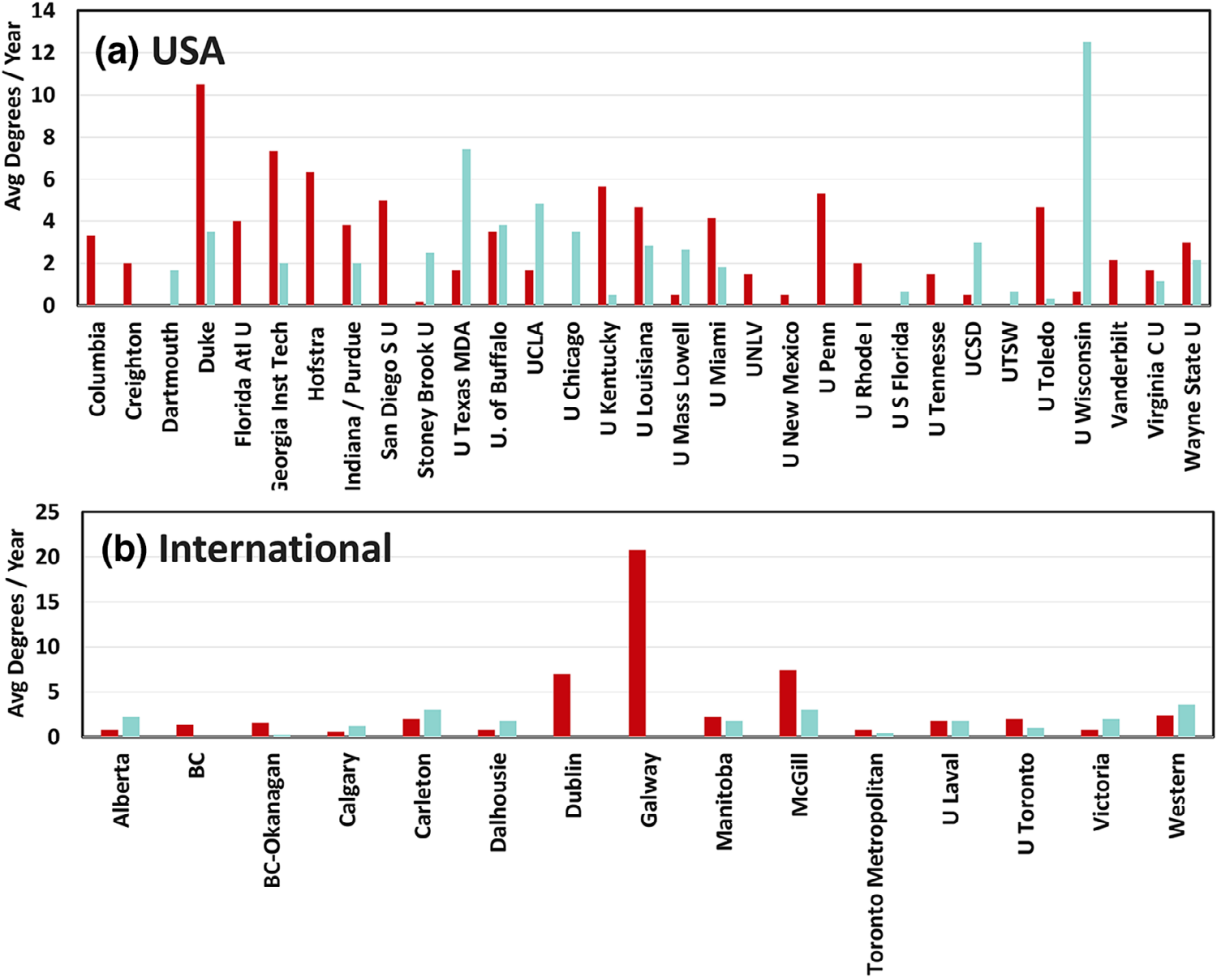
The average data over 5 years for MS and PhD graduating student numbers are shown here for (a) domestic USA institutions and (b) Canadian & Irish universities.

The survey of data started with the required self‐reporting data from all documented CAMPEP educational programs to understand the distribution of students across institutions and countries. This consisted of 59 programs, and at least 22 did not have complete data online, but the program chairs were contacted to be given a chance to provide this information. The majority of those contacted provided the data required to complete this survey. Of this total group, 46 programs had complete non‐zero data, with our total survey consisting of 31 domestic USA programs, 13 Canadian programs, and 2 Irish programs. Of the 59 programs, three did not provide us with any numbers, and two others reported the total number of graduates per year without distinction for MS or PhD. The number of students per year fluctuated significantly from year to year, so a mean of the available 7 years (2017–2023) is reported here, plotted in Figures 1a for US‐based universities and Figure 1b for non‐US‐based universities.

### 3.2 Annual trends in degrees cumulative across medical physics

**FIGURE 2:**
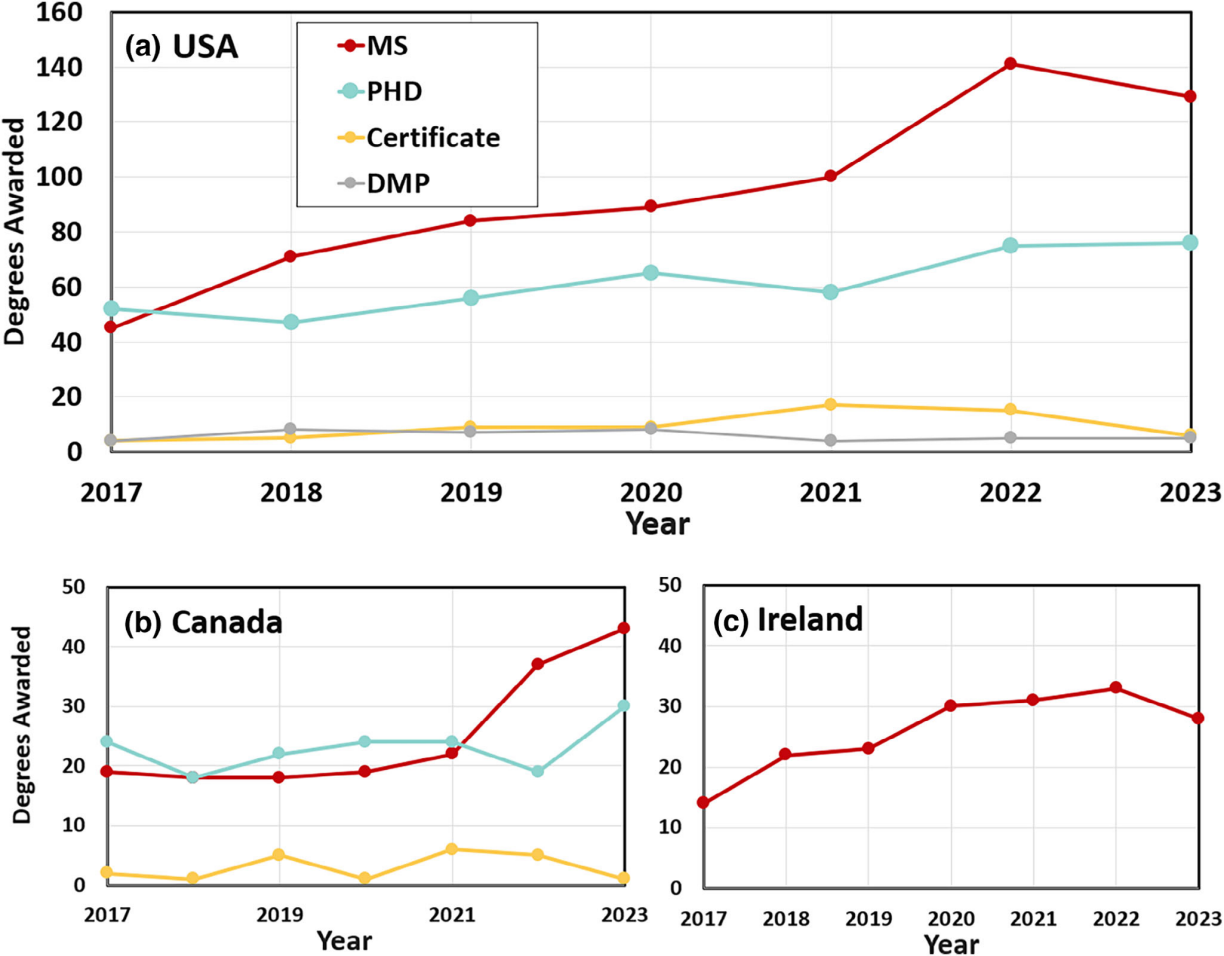
Annual trends shown for graduates from MS, PhD, Certificate, and DMP programs for (a) domestic USA programs, (b) Canadian programs, and (c) Irish programs.

The temporal trends of MS, PhD, Certificate, and DMP programs are from cumulative self‐reported statistics, plotted for the available 7 years of data from 2017 to 2023. Data from 2023 was available for nearly all programs, and updates from some programs were provided by email contact with the program directors. This annual temporal data is plotted in Figure 2.

For domestic US programs, the trend for MS graduate growth is apparent, with an average growth of 17.7 new MS graduates per year over this time, while there was a growth of PhD graduates was 3.6 per year. Certificate programs added an average of 1 graduate per year, while the DMP programs were unchanged in average numbers. It might also be noted that the 2023 numbers could be an underestimation, as several programs were not fully updated at the time of this survey.

For non‐US‐based programs, the MS growth is dominated by programs in Ireland, at National University of Ireland Galway (accredited in 2015) and University College of Dublin (accredited in 2020), which have been adding students with accreditation. Growth in Canada appears to be more about the time at which they received CAMPEP accreditation than a trend in program acceptance changes and given the smaller numbers, it is harder to assess a trend.

### 3.3 Faculty involvement in medical physics degree programs

**FIGURE 3:**
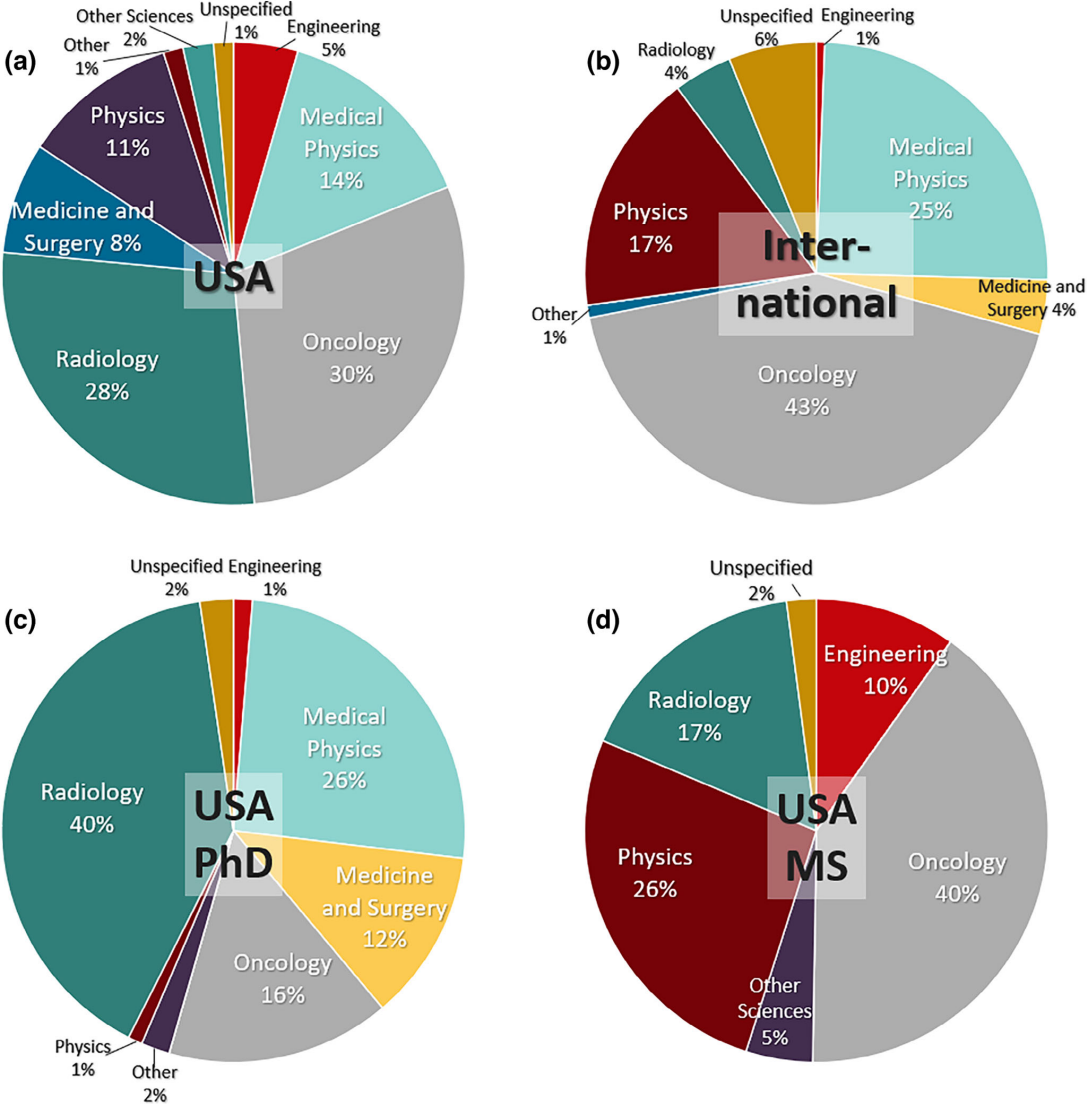
The faculty involvement in programs was separated by their home departments, graphed as % pie charts for (a) domestic USA program faculty and (b) Canadian & Irish program faculty. For the USA data, it was further separated into (c) those programs dominated by MS programs and (d) those dominated by PhD programs.

The numbers of participating faculty were compiled and graphed based upon a grouping of their home department, for the cumulative CAMPEP accredited graduate programs, as shown in Figure 3. The first two graphs are separated for (a) US‐based and (b) non‐US‐based programs, irrespective of degree makeup. Then the data for US domestic programs are shown sub‐divided out for (c) predominantly PhD programs (as determined by >2 PhD graduates per year with more PhD than MS graduates) and (d) predominantly MS programs (as determined by >2 MS graduates per year with more MS than PhD graduates).

### 3.4 Student support mechanisms in accredited medical physics degree programs

Data was made available from CAMPEP for cumulative analysis on self‐reported program funding of students. This was found from a survey of the graduate program directors with a series of questions.

**FIGURE 4:**
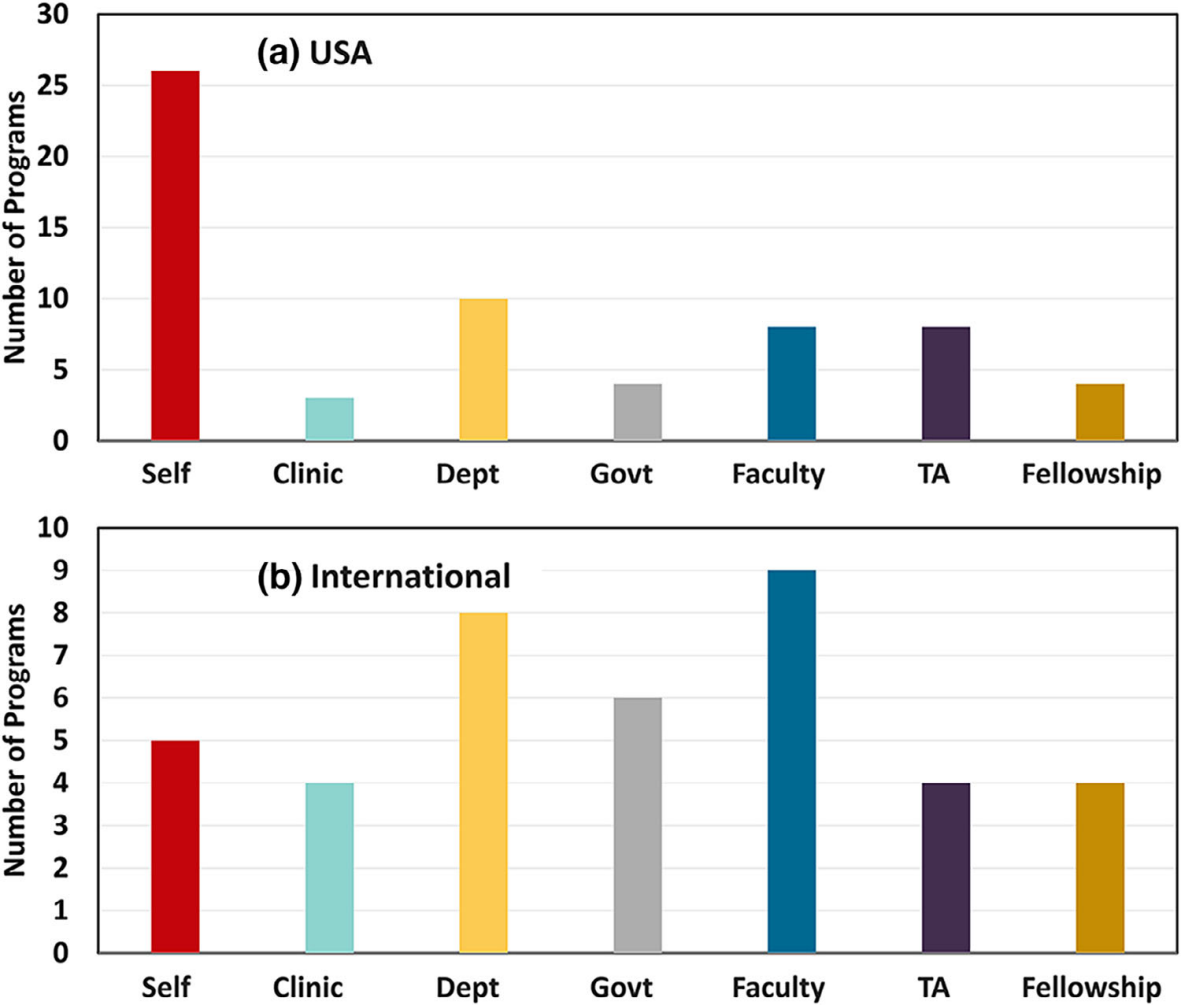
Listing of aggregate support mechanisms for MS students from CAMPEP program survey is shown, unweighted by any other factors, for (a) US‐based programs, (b) non‐US international programs.

The data are not quantitative, but rather lists ways that students are funded in each program, without percentages for each nor specificity as to exactly which programs and what number of students. As a result, it is hard to make global conclusions from this data. Nonetheless, a cumulative graph showing the fractional components of support for MS students is shown in Figure 4 across the programs

### 3.5 NIH funding trends to related departments

**FIGURE 5:**
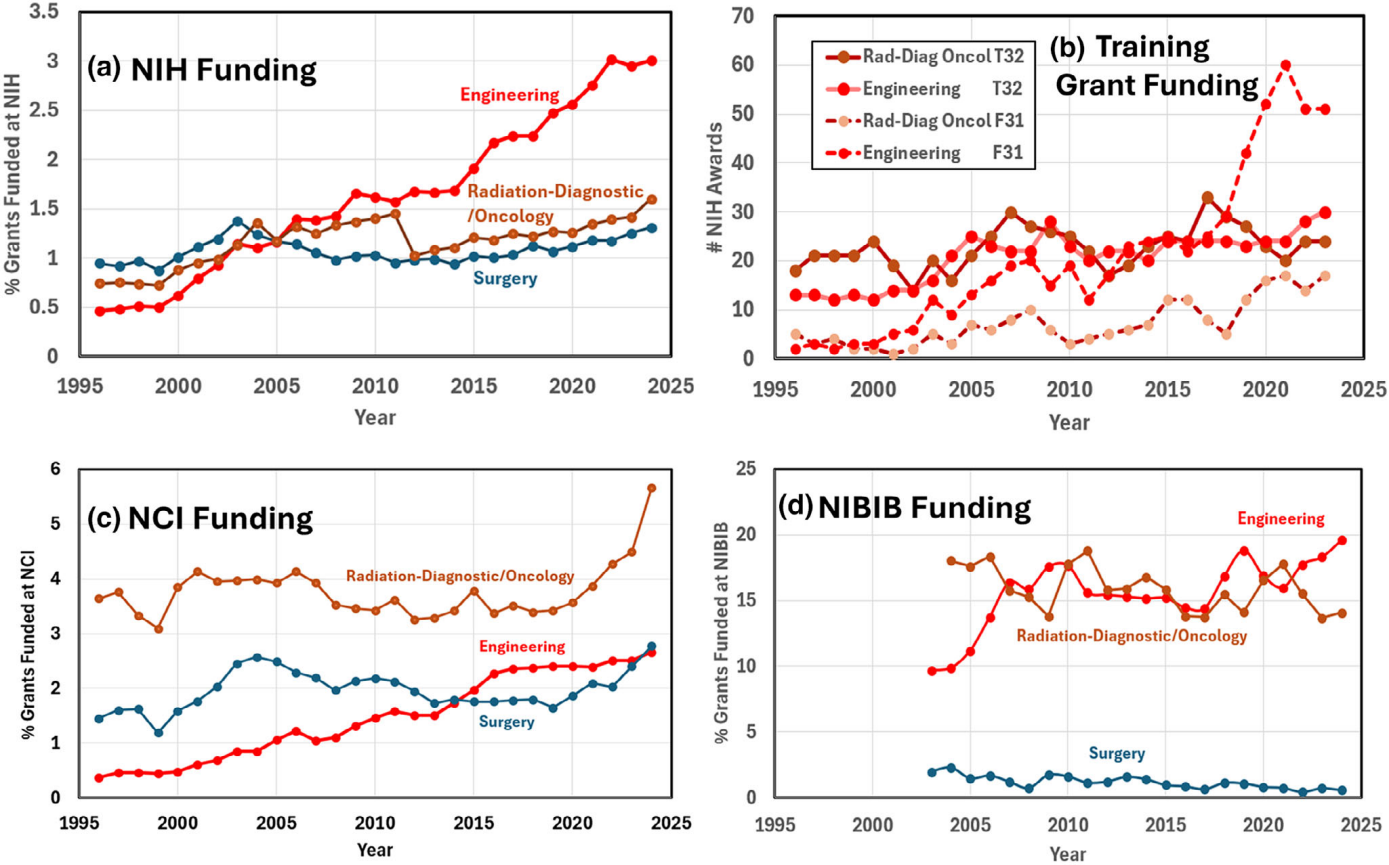
Annual NIH funding trends reported by NIH RePORTER for three department categories, (i) Radiation‐Diagnostic/Oncology, (ii) Engineering, and (iii) Surgery. These are graphed as (a) a % of all NIH grants, (b) for student training support grants, institutional T32 and individual F31, (c) a % of all NCI grants, and (d) NIBIB grants.

The annual trends in grant funding are tabulated in Figure 5 for three representative departments that are listed in NIH RePORTER, classified as (i) Radiation‐Diagnostic/Oncology dept, (ii) Engineering and Physics together, and with (iii) Surgery is included as a comparator department. The % of grants funded scales linearly with dollars of funding, and so the data is shown as % of grants funded on the *y*‐axis. The three relevant data pools for education were assessed as (a) all NIH funding, (b) National Cancer Institute (NCI) funding, (c) student training grants funding, and (d) NIBIB funding.

Figure 5a shows all NIH grants, with the obvious trend of large growth in Engineering from 0.5% of all NIH grants in 1995 to now 3% of all grants in 2024. Interestingly, this appears to be stabilized in the most recent range of 2022–2024. As a comparison, the Radiation‐Diagnostic/Oncology listing grants had smaller growth, from 0.8% in 1995 up to now 1.6% in 2024. As a comparator, Surgery grant funding went from 0.95% in 1995 to 1.35% in 2024. Interestingly the trend of all 4 groups is upward in the last 10 years, although the slope of Engineering (+0.13 %/year) is 4X higher than those for Radiation (0.038%/year) and Surgery (0.032%/year). This is a clear indicator of the trend in higher education to the expansion of biomedical engineering research programs across the country during this time. Presumably, medical physics research programs are embedded within the Radiation‐Diagnostic/Oncology department funding listing, given that most faculty (see Figure 3) are embedded within these two departments (Radiation Oncology and Radiology). Thus, the research funding increase for medical physics programs during this time period has been modest, in agreement with previous studies.^12^

However, given that Medical Physics is heavily dominated by radiation systems used in cancer, the grants funded by NCI were also examined, as shown for the same three programs in Figure 5c. In this case, the funding for Radiation‐Diagnostic/Oncology is considerably higher, at a stable average of about 3.8% of all NCI, but with a notable rise to 5.6% in 2024. In this case, Surgery NCI funding was stable near 2% average, but again with a slight rise in the latter couple of years to 2.8% by 2024. Engineering NCI funding % was a slow continuous rise throughout this period, reaching 2.7% by 2024.

In Figure 5b, the training grants are listed with institutionally obtained training grants T32 listed, and individual‐support F31 training grants. The funding growth in T32 programs is quite similar in the two groups of departments, Engineering and Radiation, with perhaps more variation in the latter. On average, their trajectories do not look that different. However, in the F31 grants, there has been a strong increase in Engineering students gaining this funding since about 2018, increasing from about 25 to about 50 today. The funding in the Radiation area has grown from near zero in 1995 to about 18 per year in 2024. The funding of grants related to Radiation‐Diagnostic/Oncology and Engineering has fluctuated but remains roughly constant and equal with each other.

## 4 DISCUSSION

With the ongoing expansion of Medical Physics as a discipline, and the nature of the education programs feeding this being a niche area, it is critical to understand how education and training are affecting the growth of the field.^2^, ^3^, ^4^ The institutional decision making by faculty largely determines the free market forces of education supply. The analysis of this public data provides a window into these trends.

### 4.1 Graduate student numbers and growth

Based on the data in Figure 1, there is a wide range of program sizes and MS/PhD ratios across institutions. About 17 institutions appear focused predominantly on the MS degree (as measured by >2 per year with more MS than PhD graduates), while 8 appear more focused on PhD program (as measured by >2 per year with more PhD than MS graduates). About 4 have an equal balance of MS and PhD graduates. Inferring any solid conclusions about international programs for MS and PhD is challenging because of the lack of data about the extent of accredited and non‐accredited programs and their participation with CAMPEP.

Perhaps the most important feature seen in this data is the temporal trend of MS graduates in the past 7 years. The number of MS graduates outpaced the number of PhD graduates in 2018, and today there are twice as many MS graduates as PhD graduates. This trend is continuing with a growth of about 17.7 new MS graduates each year as compared to a growth of only 3.6 additional PhDs per year, in the USA programs. The number of certificate graduates has grown by about 1 additional person per year, while the DMP program has shown no growth. So, certificate programs remain a small but growing segment of the CAMPEP education pathway, while DMP programs have stayed small but stable.

Outside of the US, similar but smaller trends are seen in the Canadian system, with MS growth occurring substantially in the last 2 years, now surpassing PhD programs. In Ireland, only MS programs are charted, and so this is likely more a limitation of the system and data available than anything else, but there is growth, doubling in number from 2017 to 2022.

Certificate programs are a small but growing part of the training landscape, as observed by Prisciandaro et al.^13^ Their numbers are nearly insignificant as compared to MS and PhD graduate numbers, but again, likely serve to diversify the backgrounds of trained clinical physicists. In comparison, DMP program graduates are quite few in number and do not appear to be growing. This may be because of limitations in pathways to fund this type of program, and competition by MS programs, which leads to a similar timeline to residency with perhaps similar career trajectories.^14^

In addition to separating between US‐based and non‐US‐based programs, analysis was completed on the established programs, defined as those which were accredited prior to 2015, and found that they saw an increase in both MS and PhD students of 11 and 2.6 per year, respectively. This means that there was significant growth from established programs, which was assisted by the addition of newly accredited programs.

This survey was not able to analyze the diversity in education programs, as this type of demographic data is not publicly available from websites; however, future studies might consider this as a needed value to ensure appropriate growth in the field.^15^

### 4.2 Departments involved in medical physics education

The typical clinical departments involved are perhaps not surprising, with 30% Oncology, 28% Radiology, 8% Medicine & Surgery, 14% Medical Physics, and 11% Physics faculty, as shown in Figure 3a. Given that the employment of the vast number of clinical medical physicists is in Radiation Oncology, followed by Radiology, these numbers seem reasonable. Participation of other departments can be for a variety of reasons to do with education focus, research capacity or institutional priorities in hiring faculty. In Ireland and Canada, they have a much smaller Radiology contingent, presumably illustrating more of a focus on therapeutic physics training.

When the USA numbers are broken into PhD dominated (Figure 3c) versus MS dominated (Figure 3d) programs, the MS dominated have a larger fraction of faculty in Oncology (40%) and Physics (26%) departments, while the PhD program dominated programs have a larger fraction of faculty in Radiology (40%) and Medical Physics (26%) departments. There is also an interestingly large fraction from Medicine & Surgery in the PhD program group (12%). These data may point to the fact that funding for PhD programs is more achieved through the Radiology, Medical physics, or Medicine/Surgery faculty, while the Oncology & Physics faculty focuses more on the revenue‐generating MS degrees for program support.

### 4.3 Student funding mechanisms

Based upon the data in Figure 4, self‐funding was the most commonly reported category from program directors in the USA, while in Canada and Ireland, their portfolio of funding tools was more equally balanced across the list of options. Self‐funding by students in MS programs is the singular most dominant pathway for funding this educational pathway. The motivations for using self‐funding in education programs by students may be mixed, but obviously are related to making the programs cost‐effective for the institution while serving the growing market need for more CAMPEP‐trained medical physicists. Programs with large undergraduate programs can afford to fund some with Teaching Assistantships, but others without these are left without institutional funding options.

Perhaps one of the most important observations in this data for the field of medical physics is the very modest growth of both numbers of PhDs (Figure 1a) and funding in Radiation Oncology and Radiology programs across the NIH (Figure 5a), at a time when growth in clinical medical physics is very strong. There is some positive information in the NCI funding in Radiation‐Diagnostic/Oncology has grown significantly in the last 5 years (Figure 5c), although the origins of this are not clear to date and may be in areas less related to medical physics, such as immunotherapy. The inclusion of engineering as a comparator program was intentional because biomedical engineering and medical physics are so close to each other in terms of people skills and research topics. The cause of the unparalleled growth in NIH funding within engineering schools is likely from deep investments in research infrastructure and faculty, which has occurred across the country in the last several decades.^16^ This has allowed for Tenure track faculty who have the infrastructure, financial resources, mandate, and support to carry out research at 50% or greater FTE, which has contributed to the potential for substantial NIH funding. Importantly, this differentiation shows up in training grant data too, where the numbers are slowly rising and steady in Radiation‐Diagnostic/Oncology but have a significant peak in recent years in engineering (Figure 5b). More attention to training students and mentoring faculty in grant writing to gain potential funding in F31s and T32s would benefit the field and provide needed funding support.

Increasing numbers of MS students being trained and the significant increase in need for medical physicists nationwide likely means that the fractional footprint of research has been decreasing in the field due to large clinical growth.^17^ This seems to match the trends in research versus clinical service across the country, as healthcare systems grow more in clinical practice. Research has stayed more centralized to major academic medical centers that can afford to support the infrastructures and tenure track lines that support research careers and students. It is also noteworthy that the funding in biomedical engineering has seen substantial growth, and it is likely that PhD growth is occurring much more outside of CAMPEP accredited programs. There are T32 training programs in imaging sciences and biomedical imaging which are outside of the accredited purview of CAMPEP. Some analysis of this in future studies is warranted to see if the field of medical physics is losing its research footprint to non‐medical physics programs.

CAMPEP survey data on funding mechanisms for student support were made available as well. This was survey data provided voluntarily from individual program directors, listing the general categories of funding mechanisms available to programs. While this data is imperfect because it is qualitative rather than quantitative, and there are overlapping and complementary information sets without definite numbers of students or percentages of the program, but it was still used as a tool to assay the funding of master's and doctoral programs as a pool.

A study published in 2020 analyzed the number of NIH grants awarded to members of the AAPM and discussed that from 2002–2019, the number of NIH grants awarded to members of the AAPM has dropped from 2.9% to 2.1%.^12^ The grants awarded were sorted by the study's authors into five categories: imaging, therapy, image‐guided therapy, educational, and other. Imaging grants were those that predominantly used language that referred to imaging modalities such as magnetic resonance imaging or computed tomography. Therapy grants were those that used language that referred to therapeutic treatments such as radiation therapy or brachytherapy. Image‐guided therapy grants were those that used terminology that was related to both imaging and therapy. During the timeframe of the study, the vast majority of grants awarded to AAPM members were classified as imaging grants, often being in excess of 60% of all grants analyzed. The second most prevalent grant classification was image‐guided therapy, which grew over the course of the study from 13% in 2002 to 27% in 2019. During the same time frame, the portion of therapy‐related grants remained stable and was 6% in both 2002 and 2019.

Despite most AAPM‐member grants going to imaging‐classified grants, the grants going to AAPM members are a small fraction (<10%) of all NIH grants awarded for Imaging research. Meanwhile AAPM members received a much larger portion of all therapy related grants. This points to more money from NIH grants going towards imaging research than therapy research which may contribute to the number of faculty whose primary appointment is in radiology, especially in PhD programs.

## 5 SUMMARY

This survey of public data provided a useful perspective on the status and trends in Medical Physics education. From 2017 to 2023, there has been consistent growth in MS graduates, increasing at an average of 17.7 per year, while the growth in PhDs has been slower at 3.6 per year. By 2025, there will be double the number of MS graduates as PhD graduates. As the field of medical physics grows, this survey suggests that growth in the field of medical physics education is and will be dominated by MS graduates. These programs which focus on larger MS graduating numbers and have a large fraction of self‐funded students in domestic programs. Interestingly, non‐US‐based MS and US‐based PhD programs have a broader range of financial support mechanisms. Additionally, those programs dominated by MS education graduates have their largest fraction of faculty in Radiation Oncology departments, followed by Physics departments, whereas those programs dominated by PhD graduates have their largest fraction of faculty in Radiology departments, followed by Medical Physics departments. Overall, NIH funding in the space of Radiation‐Diagnostic/Oncology has been slowly growing over this timeframe, but with a notable recent rise in NCI funding in the last 5 years. However, there has been a substantial 5X‐6X in NIH funding for engineering research programs during this time, including significant increases in F31 and T32 training grants. The origins of these trends are not entirely clear from this survey, but it seems as though the research funding that supports PhD training in medical physics is likely to be missing the ongoing NIH funding growth trend in biomedical engineering. The overall picture presented in this data is one where the field of medical physics is growing solidly as a clinical discipline, with a somewhat static research component in terms of size and support.

## AUTHOR CONTRIBUTIONS

Brian W. Pogue conceived of the study, analyzed the data, and drafted the manuscript. Alexander P. Niver collected all data in the study, analyzed the data, and edited the manuscript.

## CONFLICT OF INTEREST STATEMENT

The authors have no conflicts of interest relevant to the content of this paper.
